# Porcine RIG-I and MDA5 Signaling CARD Domains Exert Similar Antiviral Function Against Different Viruses

**DOI:** 10.3389/fmicb.2021.677634

**Published:** 2021-06-11

**Authors:** Shuangjie Li, Qi Shao, Yuanyuan Zhu, Xingyu Ji, Jia Luo, Yulin Xu, Xueliang Liu, Wanglong Zheng, Nanhua Chen, François Meurens, Jianzhong Zhu

**Affiliations:** ^1^Comparative Medicine Research Institute, Yangzhou University, Yangzhou, China; ^2^College of Veterinary Medicine, Yangzhou University, Yangzhou, China; ^3^Joint International Research Laboratory of Agriculture and Agri-Product Safety, Yangzhou, China; ^4^Jiangsu Co-innovation Center for Prevention and Control of Important Animal Infectious Diseases and Zoonoses, Yangzhou, China; ^5^BIOEPAR, INRAE, Oniris, Nantes, France; ^6^Department of Veterinary Microbiology and Immunology, Western College of Veterinary Medicine, University of Saskatchewan, Saskatoon, SK, Canada

**Keywords:** RLRs, porcine, CARD, signaling activity, antiviral function, transcriptome

## Abstract

The RIG-I-like receptors (RLRs) RIG-I and MDA5 play critical roles in sensing and fighting viral infections. Although RIG-I and MDA5 have similar molecular structures, these two receptors have distinct features during activation. Further, the signaling domains of the N terminal CARD domains (CARDs) in RIG-I and MDA5 share poor similarity. Therefore, we wonder whether the CARDs of RIG-I and MDA5 play similar roles in signaling and antiviral function. Here we expressed porcine RIG-I and MDA5 CARDs in 293T cells and porcine alveolar macrophages and found that MDA5 CARDs exhibit higher expression and stronger signaling activity than RIG-I CARDs. Nevertheless, both RIG-I and MDA5 CARDs exert comparable antiviral function against several viruses. Transcriptome analysis showed that MDA5 CARDs are more effective in regulating downstream genes. However, in the presence of virus, both RIG-I and MDA5 CARDs exhibit similar effects on downstream gene transcriptions, reflecting their antiviral function.

## Introduction

The innate immune system acts as the first line of host defense, sensing various pathogens by detecting the pathogen-associated molecular patterns (PAMPs) through its pattern recognition receptors (PRRs) (Takeuchi and Akira, 2010). RIG-I-like receptors (RLRs) form a family of DExD/H-box helicases, including homologous RIG-I, MDA5, and LGP2. They play key roles in the detection of cytosolic RNA and the cellular antiviral activity (Zou et al., 2009). RIG-I and MDA5 have similar domain architecture: The N-terminal signal-effective two-tandem caspase recruitment domains (CARDs), the DExD/H-box helicase domain, and the C-terminal repressor domain (CTD) or regulatory domain (RD) (Schlee, 2013; Chen et al., 2017). Due to lack of N terminal CARDs, LGP2 has no signaling activity (Li et al., 2009).

In spite of the structural similarity, RIG-I and MDA5 display different features. First, the optimal ligands are different. The best RNA recognized by RIG-I is the blunt ended 5′-ppp double-stranded RNA (dsRNA), whereas MDA5 prefers the long dsRNA with no end specificity (Reikine et al., 2014; Rehwinkel and Gack, 2020). Second, the activation mechanisms of RIG-I and MDA5 are distinct. RIG-I CARDs are tetramerized into signaling effective stable lock-washer structure through K63-ubiquitination/polyubiquitin binding of the CARDs and/or the filament formation by RIG-I RD-helicase along the short dsRNA chain, whereas MDA5 CARDs form a tetramer structure mainly through the filament formation along the long dsRNA by RD-helicase (Sohn and Hur, 2016; Chen et al., 2017). Third, RIG-I and MDA5 differ in sensing viruses. RIG-I mainly senses negative-sense single-stranded RNA viruses generating short 5′-ppp dsRNA during their replication, which is consistent with the short RNA agonists. In contrast, MDA5 senses positive-sense single-stranded RNA viruses such as the *Picornaviridae* (Chen et al., 2017; Brisse and Ly, 2019).

As the signaling domains, RIG-I and MDA5 CARDs, upon forming tetramers, recruit downstream common adaptor MAVS on the mitochondrial and peroxisomal membranes via homophilic interactions, which aggregates into the prion-like signaling complex (Wu and Hur, 2015; Sohn and Hur, 2016). In turn, MAVS signalosomes further activate TRAF3/TBK1/IKKε and TRAF6/IKKs, signal to transcription factors IRF3 and NF-κB, and drive IFN and proinflammtory cytokine expression, enabling the cells to achieve antiviral status (Wu and Hur, 2015). During the comparative study on porcine RIG-I and MDA5, we noticed that the RIG-I and MDA5 CARDs have poor amino acid similarity (<20%), which triggered our interest. We wondered whether these two types of CARDs have similarities in cell signaling and antiviral activity. Our study showed that porcine RIG-I and MDA5 CARDs exert similar antiviral functions despite the disparity in signaling strength when considered alone.

## Materials and Methods

### Cells, Reagents, and Viruses

HEK293T and Vero cells were cultured in DMEM (Hyclone Laboratories, United States) containing 10% fetal bovine serum (FBS) and 100 IU/mL of penicillin plus 100 g/mL streptomycin. Porcine alveolar macrophages (PAMs, 3D4/21) were cultured in RPMI (Hyclone Laboratories) containing 10% FBS with penicillin/streptomycin. All cells were maintained at 37°C with 5% CO2 in a humidified incubator. TRIpure Reagent for RNA extraction was from Aidlab (Beijing, China). HiScript^®^ 1st Strand cDNA Synthesis Kit, ChamQ Universal SYBR qPCR Master Mix, and 2 × Taq Master Mix (Dye plus) were from Vazyme Biotech Co., Ltd. (Nanjing, China). Gateway^®^ LR Clonase^TM^ II Enzyme mix was from Thermo Fisher Scientific (Shanghai, China). TransIT-LT1 Transfection Reagent was purchased from Mirus Bio (Madison, United States). Anti-HA mouse mAb (HT301), anti-GFP mouse mAb (HT801), anti-Actin mouse mAb (HC201), HRP anti-mouse IgG (HS201), HRP anti rabbit IgG (HS101), and the TransDetect Double-Luciferase Reporter Assay Kit were bought from TransGen Biotech (Beijing, China). The anti-p-TBK1 (D52C2) (5483S), anti-TBK1 (D1B4) (3504), anti-p-IRF3 (4D4G) (29047S), anti-IRF3 (D614C) (11904T), and anti-HA (C29F4) (3724S) rabbit mAb were all from Cell Signaling Technology (Danvers, MA, United States). Donkey Anti-Rabbit IgG Alexa Fluor 647 (ab150075) were from Abcam (Shanghai, China). The anti-Influenza A NS1 mouse mAb (sc-130568) were purchased from Santa Cruz Biotechnology (Dallas, TX, United States). The viruses HSV-1-GFP, VSV-GFP, SeV-GFP, and EMCV were used as we previously reported (Li et al., 2020). Influenza virus H9N2 and PR8 H1N1 were provided by Dr. Hongjun Chen at Shanghai Veterinary Research Institute China.

### Molecular Cloning

The two CARD domains (CARDs, N terminal 600 bp in length for coding of N-200 amino acids) from porcine RIG-I and MDA5 were amplified by PCR from the porcine RIG-I and MDA5 pcDNA expression plasmids, respectively, which we prepared before in our laboratory. The PCR primers for amplification of both CARDs are available upon request. The PCR products were subjected to restriction enzyme digestion and subcloned into *Sal*I*/Eco*RV sites of the Gateway entry vector pENTR4-2HA, which carries a 2HA tag to express C-terminal HA tagged proteins. The sequence confirmed pRIG-I-CARDs and pMDA5-CARDs were transferred from pENTR4 vectors to Destination vectors pDEST47 (Addgene) or pLenti CMV Puro DEST (Addgene) by LR recombinations to obtain the final pcDNA or lentiviral recombinant expression vectors, which were named as pcDNA pRIG-I/pMDA5 CARDs or pLenti pRIG-I/pMDA5 CARDs.

### Western Blotting

The cells in the 24-well plate (2–3 × 10^5^ cells/well) or 12-well plate (5 × 10^5^ cells/well) were transfected for 24 h and then infected (or not) with different viruses. Cells were harvested and lysed in 50 μL or 100 μL RIPA buffer (50 mM Tris pH 7.2, 150 mM NaCl, 1% sodium deoxycholate, 1% Triton X-100) containing protease inhibitors on ice for 30 min. The cell lysates were boiled with 1 × SDS sample buffer at 100°C for 5–10 min, and then the cleared lysates were resolved on 8–12% SDS-polyacrylamide gels. The protein bands on gels were transferred onto PVDF membranes and the membranes blocked with 5% non-fat dry milk Tris-buffered saline, pH 7.4, with 0.1% Tween-20 (TBST). Next PVDF membranes were sequentially incubated with various primary antibodies and HRP-conjugated goat anti-mouse or rabbit IgG. The protein signals were detected with enhanced chemiluminescence (ECL) substrate (Tanon, China) and visualized by Western blot imaging system (Tanon, China).

### Fluorescence Microscopy

Porcine alveolar macrophages grown on a 15 mm glass bottom cell culture dish (Cat No: 801002, NEST, China, 5 × 10^5^ cells) were transfected with 0.75 μg pRIG-I-HA, pRIG-I-CARDs-HA, pMDA5-HA, and pMDA5-CARDs-HA expression plasmids for 24 h, respectively, using TransIT-LT1 Transfection Reagent. Then, the cells were fixed with 4% paraformaldehyde at room temperature (RT) for 30 min, and permeabilized with 0.5% Triton X-100 for 20 min. After washing with PBS, the cells were sequentially incubated with primary anti-HA rabbit pAb (1:200), and secondary antibody Donkey Anti-Rabbit IgG Alexa Fluor 647 (1:500). The stained cells were counter-stained with 0.5 μg/mL 4′,6-diamidino-2-phenylindole (DAPI, Beyotime, China) at 37°C for 15 min to stain the cell nucleus. Lastly, PAMs were visualized under laser-scanning confocal micorscope (LSCM, Leica SP8, Solms, Germany) at the excitation wavelength of 647 nm.

Porcine alveolar macrophages grown on a 24-well plate (2 × 10^5^ cells/well) were transfected with 0.5 μg pRIG-I-2CARD and pMDA5-2CARD plasmids, respectively, using TransIT-LT1 Transfection Reagent. A total of 24 h later, the transfected cells were infected with various GFP viruses. Then the GFP signal was visualized under Fluorescence microscope (Olympus Corporation, IX53, Japan).

### Promoter Driven Luciferase Reporter Gene Assays

Porcine alveolar macrophages grown in 96-well plates (2 × 10^4^ cells/well) were co-transfected by TransIT-LT1 Transfection Reagent with ISRE-firefly luciferase reporter or ELAM (NF-κB)-firefly luciferase (Fluc) reporter (10 ng/well) and β-actin Renilla luciferase (Rluc) reporter (0.2 ng/well), together with the pRIG-I-CARDs, pRIG-I, pMDA5-CARDs, pMDA5 expressing pcDNA plasmids or vector control (20 ng/well), which was normalized to 50 ng by empty pcDNA vector. About 24 h post-transfection, the cells were harvested and lysed, and both Fluc and Rluc activities were sequentially measured using the TransDetect Double-Luciferase Reporter Assay Kit. The results were expressed as fold inductions of ISRE or ELAM (NF-κB)-Fluc compared with that of vector control after Fluc normalization by corresponding Rluc.

### Quantitative RT-PCR

Porcine alveolar macrophages grown in 24-well plates (2–3 × 10^5^ cells) were subjected to different treatments. The treated cells were harvested and RNA extracted with TRIpure Reagent. The extracted RNA was reverse transcribed into cDNA with HiScript^®^ 1st Strand cDNA Synthesis Kit, then the target gene expressions were measured by quantitative PCR with ChamQ Universal SYBR qPCR Master Mix using StepOne Plus equipment (Applied Biosystems). The qPCR program is denaturation at 95°C for 30 s followed by 40 cycles of 95°C for 10 s and 60°C for 30 s. The qPCR primers for pIFNβ, pISG56, pTNF-α, pβ-actin, H9N2-HA, H9N2-M, H1N1-M, HSV-1, VSV, EMCV, and GFP are shown in Table 1. The transcriptional levels of target genes were quantified using the ΔΔCT calculation method.

**TABLE 1:** Primers for RT-qPCR in this study.

**Primer names**	**Primer sequences**	**Length**
pIFNβ	F: tgagcattctgcagtacctga	116 bp
	R: ccggaggtaatctgtaagtctgt	
pISG56	F: atgggagttggtcattcaaga	127 bp
	R: caggtgtttcacataggcca	
pTNFα	F: atcgccgtctcctaccaga	147 bp
	R: tcgatcatccttctccagct	
pβ-actin	F: atgaagatcaagatcatcgcg	116 bp
	R: tcgtactcctgcttgctgatc	
SIV H9N2 HA	F: aaaccaatgatagggccaa	204 bp
	R: ttgcactacacagttaccac	
SIV H9N2 M	F: gaccratcctgtcacctctgac	106 bp
	R: agggcattytggacaaakcgtcta	
HSV1 gB	F: ttctgcagctcgcaccac	295 bp
	R: ggagcgcatcaagaccacc	
VSV Glycoprotein	F: gaggagtcacctggacaatcact	121 bp
	R: tgcaaggaaagcattgaacaa	
EMCV Polyprotein	F: tcaccgtgaagtccggcagt	134 bp
	R: tgtcagacgctgtggcctga	
H1N1 M	F: atcctgtcacctctgactaaggg	80 bp
	R: tctacgctgcagtcctcgc	
SeV GFP	F: gcaacatcctggggcacaagct	246 bp
	R: cgcgcttctcgttggggtcttt	

*p denotes porcine.*

### Plaque Assays

Vero cells in 12-well plates (8 × 10^5^ cells) were grown into monolayer, and then infected for 2 h with the tenfold serially diluted cell supernatants (0.4 mL/well) from virus infected PAMs. The infected Vero cells were washed and overlaid with an appropriate volume of immobilizing medium constituted 1:1 mixture of warmed 2 × DMEM with 4% FBS and a stock solution of heated 1.6% low melting agarose. Plaque formation could take 1–3 days depending on the viruses being analyzed. The immobilizing medium were discarded by tipping and cells were fixed and stained with crystal violet staining solution (0.05% w/v crystal violet, 1% formaldehyde, 1 × PBS and 1% methanol) for 1 h at RT. After staining, cells were washed with tap water until the clear plaques appeared. The plaques were counted, and photos were taken.

### Transcriptome Analysis

Porcine alveolar macrophages grown in 6-well plates (5 × 10^5^ cells) were transfected with 1 μg pDNA pRIG-I-CARDs and pcDNA pMDA5-CARDs plasmids for 24 h, respectively, using TransIT-LT1 Transfection Reagent, and they were then infected with 0.01 MOI swine influenza virus H9N2 for 12 h or added PBS as a control. The treated cells were harvested, and RNAs were extracted for Transcriptome sequencing. Transcriptome analysis was performed in Novogene (Beijing, China). Briefly, a total amount of 1 μg RNA per sample was used for the RNA sample preparations. Sequencing libraries were generated using NEBNext^®^ UltraTM RNA Library Prep Kit for Illumina^®^ (NEB, United States). After cluster generation, the library preparations were sequenced on an Illumina Novaseq platform and 150 bp paired-end reads were generated. The raw data were processed to remove reads containing adapters, reads containing ploy-N, and low-quality reads to obtain clean reads. The paired-end clean reads were aligned to the reference genome using Hisat2 v2.0.5, and the read numbers mapped to each gene were counted using FeatureCounts v1.5.0-p3. FPKM (the number of Fragments Per Kilobase of transcript sequence per Millions base pairs sequenced) of each gene was calculated based on the length of the gene to estimate gene expression levels. Based on the FPKM, differential expression analysis of two conditions was performed using the edgeR R package (3.18.1). The *P*-values were adjusted using the Benjamini and Hochberg method, and those with a corrected *P*-value of 0.05 and absolute fold change of 2 were set as the threshold for significantly differential expression.

### Statistical Analysis

All the experiments are representative of three similar experiments and the representative experimental data in graphs were shown as the mean ± SD of duplicate wells. The statistical analysis was performed with a *t-*test or a one-way ANOVA where appropriate, and both are built within the software GraphPad Prism 6.0.

## Results

### The Expression and Signaling Activity of Porcine RIG-I and MDA5 CARDs

**FIGURE 1:**
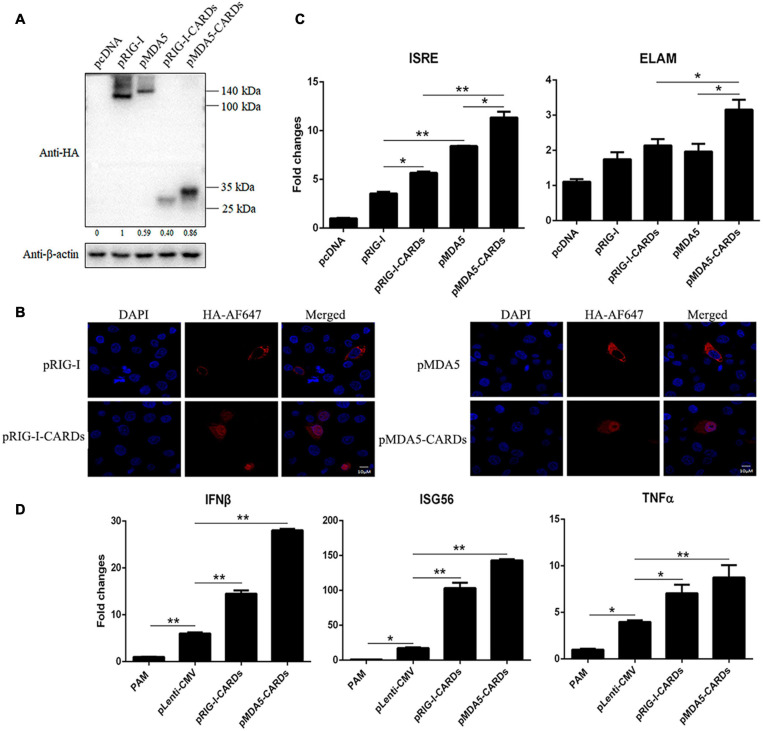
The protein expression, cellular localization and signaling activity of porcine RIG-I and MDA5 CARDs. **(A)** The pRIG-I-HA, pMDA5-HA, pRIG-I-CARDs-HA, pMDA5-CARDs-HA, and vector pcDNA (0.5 μg each) were transfected into 293T cells in 24-well plates (3 × 10^5^ cells/well) for 24 h using Lipofectamine 2000 (Thermo Fisher Scientific). The cells samples were detected by Western-blotting with anti-HA and anti-actin mAb. The densitometry values after actin normalization were shown below the blot. **(B)** PAMs grown on 15-nm glass bottom cell culture dish (5 × 10^5^ cells) were transfected with the indicated pcDNA expression plasmids (0.75 μg each) using TransIT-LT1 Transfection Reagent for 24 h. The cells were examined for localization by con-focal fluorescence microscopy. **(C)** PAMs grown in 96-well plates (2 × 10^4^ cells/well) were transfected with 20 ng indicated pcDNA expression plasmids, plus ISRE-Fluc/ELAM-Fluc (10 ng) and Rluc (0.2 ng) using TransIT-LT1 Transfection Reagent for 24 h. The luciferase activities were measured with Double-Luciferase Reporter Assay. **(D)** PAMs grown on 24-well plate (3 × 10^5^ cells/well) were transfected with pLenti-pRIG-I-CARDs-HA and pLenti-pMDA5-CARDs-HA (0.5 μg each) and pLenti-CMV vector using TransIT-LT1 Transfection Reagent for 24 h. Then the cells were analyzed by RT-qPCR for downstream gene expressions as indicated. The signs “*” and “**” denote *p* < 0.05 and *p* < 0.01, respectively.

We transfected porcine RIG-I and MDA5 CARDs together with full length porcine RIG-I and MDA5 into 293T cells, and Western blotting showed that different from the lower expression level of porcine MDA5 than RIG-I, MDA5 CARDs has higher expression than RIG-I CARDs (Figure 1A). RIG-I and MDA5 CARDs we cloned are both composed of N-terminal 200 amino acids but were expressed as about 30 kDa proteins; they were of higher molecular weights than expected, indicating existence of post-translational modifications (Figure 1A). In addition, the MDA5 CARDs exhibited a higher molecular weight than RIG-1 CARDs, suggesting different post-translational modifications (Figure 1A). Curiously, relative to cytoplasmic localization of RIG-I and MDA5 in PAMs, we found that both RIG-I and MDA5 CARDs have a nuclear localization which might contribute to their signaling activity (Figure 1B).

As the signaling domains, porcine RIG-I and MDA5 CARDs harbor higher ISRE and NF-κB promoter activity than the corresponding full length RIG-I and MDA5 (Figure 1C). Consistent with the higher signaling activity of porcine MDA5 than RIG-I, MDA5 CARDs also has significantly higher signaling activity than RIG-I CARDs (*p* < 0.05, Figure 1C). The downstream gene transcription was analyzed by RT-qPCR in CARDs transfected PAMs, and the results showed that both CARDs significantly (*p* < 0.05) induce downstream IFNβ, ISG56 and TNFα gene expressions with MDA5 CARDs inducing higher levels of gene transcription than RIG-I CARDs (Figure 1D).

### The Antiviral Function of RIG-I and MDA5 CARDs Against VSV, SeV, EMCV, and HSV-1

**FIGURE 2:**
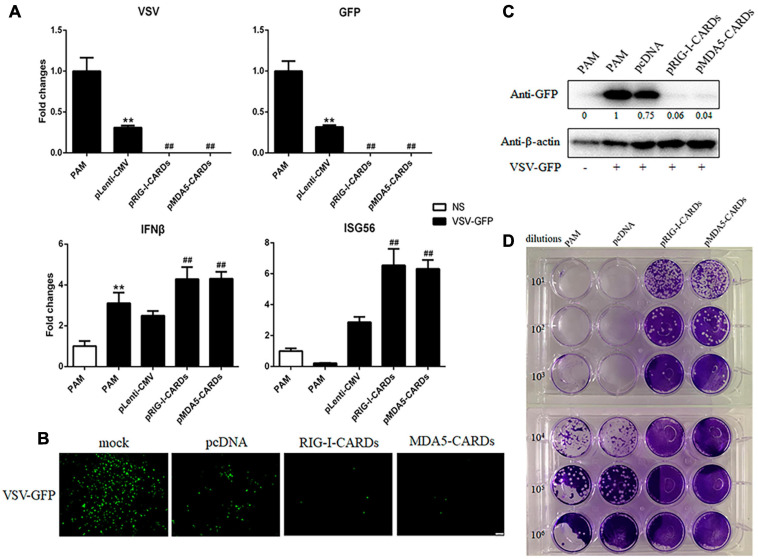
The anti-VSV activity of porcine RIG-I and MDA5 CARDs. **(A)** PAMs grown on 24-well plates (3 × 10^5^ cells/well) were transfected with pLenti-pRIG-I-CARDs-HA and pLenti-pMDA5-CARDs-HA and pLenti-CMV vector (0.5 μg each) using TransIT-LT1 Transfection Reagent for 24 h. Then the cells were infected with VSV-GFP virus at MOI of 0.01 for 12 h and analyzed by RT-qPCR for virus replication and downstream gene expressions as indicated. **(B–D)** PAMs grown on 24-well plate were transfected and infected with VSV as above. The GFP signals were visualized under microscope **(B)**. The cells samples were detected by Western-blotting with anti-GFP mAb, with the densitometry values after actin normalization shown below the GFP blot. **(C)**. The supernatants from VSV infected PAMs were subjected to infection of Vero cells and the viral plaque were observed at 24 h post infection **(D)**. **p < 0.01 vs. blank controls or mock transfection; ^##^*p* < 0.01 vs. vector controls.

**FIGURE 3:**
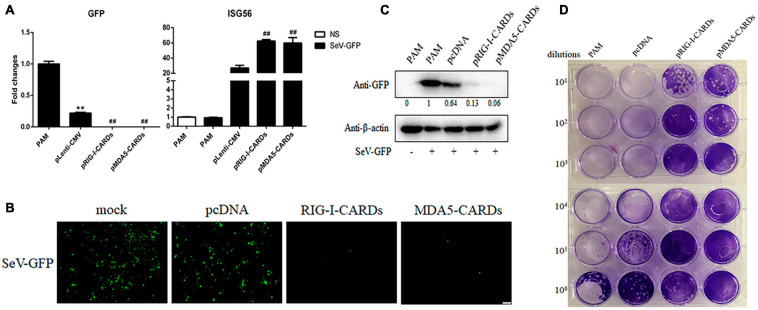
The anti-SeV activity of porcine RIG-I and MDA5 CARDs. **(A)** PAMs grown on 24-well plates (3 × 10^5^ cells/well) were transfected with pLenti-pRIG-I-CARDs-HA and pLenti-pMDA5-CARDs-HA and pLenti-CMV vector (0.5 μg each) for 24 h. Then the cells were infected with SeV-GFP virus at MOI of 0.01 for 12 h and analyzed by RT-qPCR for virus replication and downstream gene expressions as indicated. **(B–D)** PAMs grown on 24-well plate were transfected and infected as above. The GFP signals were visualized under microscope **(B)**. The cells samples were detected by Western-blotting with anti-GFP mAb, with the densitometry values after actin normalization shown below the GFP blot. **(C)**. The supernatants from SeV infected PAMs were subjected to infection of Vero cells and the viral plaque were visualized at 24 h post infection **(D)**. **p < 0.01 vs. blank controls; ^##^*p* < 0.01 vs. vector controls.

**FIGURE 4:**
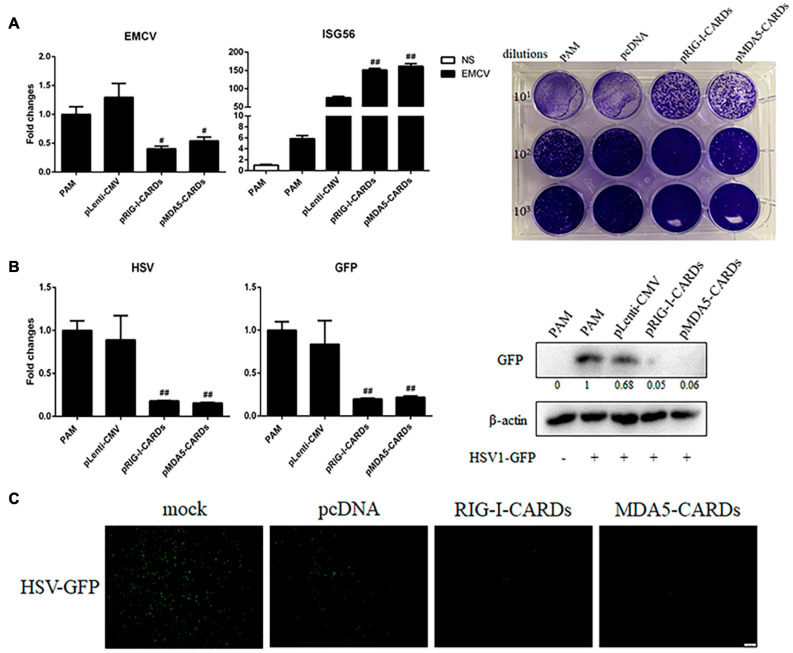
The anti-EMCV and HSV-1 activity of porcine RIG-I and MDA5 CARDs. **(A)** PAMs grown on 24-well plates (3 × 10^5^ cells/well) were transfected with the indicated CARDs expression plasmids and control vectors (0.5 μg each) using TransIT-LT1 Transfection Reagent for 24 h. Then the cells were infected with EMCV virus at MOI of 0.01 for 12 h and analyzed by RT-qPCR for virus replication, downstream gene expression and by plaque assay for observation of plaque formation in Vero cells at 24 h post infection. **(B,C)** PAMs grown on 24-well plate (3 × 10^5^ cells/well) were transfected with CARDs expression plasmids and pLenti-CMV vector (0.5 μg each) using TransIT-LT1 Transfection Reagent for 24 h. Then the cells were infected with HSV-1-GFP virus at MOI of 0.01 for 12 h. The cells samples were analyzed by RT-qPCR for virus replication and downstream gene expressions as indicated or detected by Western-blotting with anti-GFP mAb **(B)**. The GFP signals were visualized under microscope **(C)**. ^#^*p* < 0.05, ^##^*p* < 0.01 vs. vector controls.

We then assayed the antiviral functions of porcine RIG-I and MDA5 CARDs in transfected PAMs. For VSV infection, the two types of CARDs showed comparable anti-VSV activities as observed by RT-qPCR analysis of Glycoprotein and GFP mRNA expressions, WB and fluorescence detection of viral GFP, and plaque assay analysis of the virus titer (Figures 2A–D). Accordingly, the levels of IFNβ and ISG56 gene expressions were similar in the two CARDs transfected and VSV infected cells (bottom, Figure 2A). For SeV infection, the two types of CARDs showed comparable anti-SeV activity as evidenced by RT-qPCR, WB, and fluorescence detection of GFP, and plaque assay analysis of the virus titer (Figures 3A–D). Additionally, comparable ISG56 gene expression levels were induced (right, Figure 3A). For EMCV infection, the two types of CARDs triggered comparable anti-viral activity as showed by RT-qPCR detection of viral polyprotein, ISG56 gene expression, and plaque assay analysis of the virus titer (Figure 4A). Then, for HSV-1 infection, the two types of CARDs showed comparable anti-HSV-1 activity as evidenced by RT-qPCR detection of viral gB and GFP, WB, and fluorescence detection of GFP (Figures 4B,C).

### The Antiviral Function Against Influenza Viruses by CARDs and Transcriptome Analysis

**FIGURE 5:**
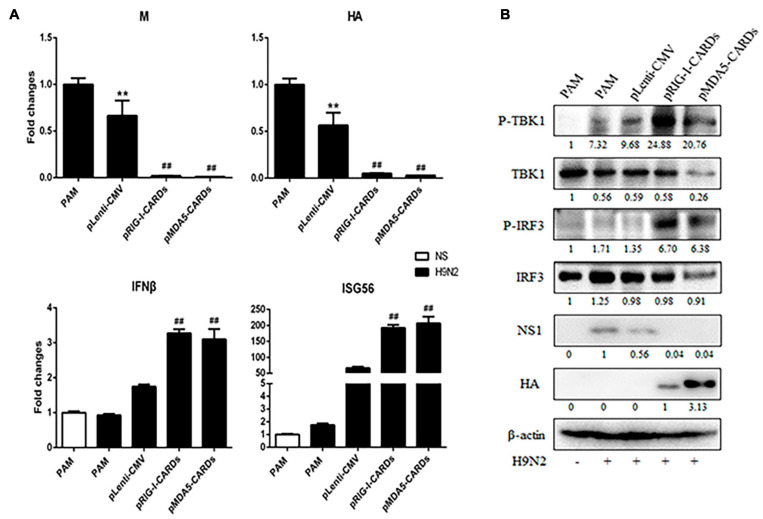
The anti-influenza virus H9N2 activity of porcine RIG-I and MDA5 CARDs. PAMs grown on 24-well plates (3 × 10^5^ cells/well) were transfected with CARDs expression plasmids and pLenti-CMV vector (0.5 μg each) using TransIT-LT1 Transfection Reagent for 24 h. Then the cells were infected with H9N2 virus at MOI of 0.01 for 12 h. The cells samples were analyzed by RT-qPCR for virus replication and downstream gene expressions as indicated **(A)** or detected by Western-blotting using the indicated antibodies **(B)**. The densitometry values after actin normalization were shown below the blots. **p < 0.01 vs. blank controls; ^##^*p* < 0.01 vs. vector controls.

**FIGURE 6:**
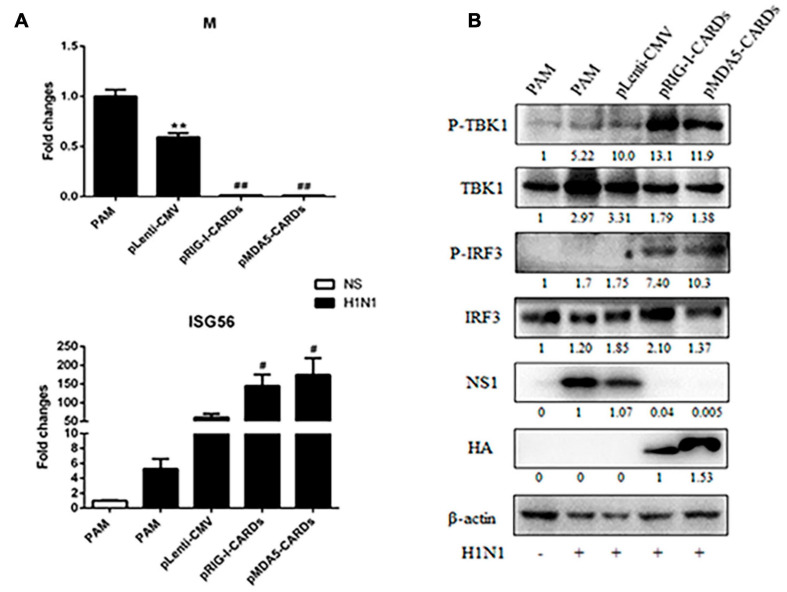
The anti-influenza virus H1N1 activity of porcine RIG-I and MDA5 CARDs. PAMs grown on 24-well plates (3 × 10^5^ cells/well) were transfected with CARDs expression plasmids and pLenti-CMV vector (0.5 μg each) using TransIT-LT1 Transfection Reagent for 24 h. Then the cells were infected with H1N1 virus at MOI of 0.01 for 12 h. The cells samples were analyzed by RT-qPCR for viral M gene and downstream ISG56 gene expressions **(A)** or detected by Western-blotting using the indicated antibodies **(B)**. The densitometry values after actin normalization were shown below the blots. **p < 0.01 vs. blank controls; ^#^*p* < 0.05, ^##^*p* < 0.01 vs. vector controls.

We further assessed the anti-influenza virus activity of the two types of CARDs. For both H9N2 and H1N1 PR8 infections, RIG-I and MDA5 CARDs induced comparable reduction of viral M and HA gene transcription and NS1 protein expression (Figures 5, 6). Upon virus infection, the upregulation of phosphorylated TBK1 was observed, whereas with the expression of CARDs, the phosphorylated IRF3 and further upregulation of phosphorylated TBK1 were obvious (Figures 5B, 6B). The cellular IFNβ and ISG56 gene expressions were also subjected to similar upregulation by the two types of CARDs (Figures 5A, 6A).

**FIGURE 7:**
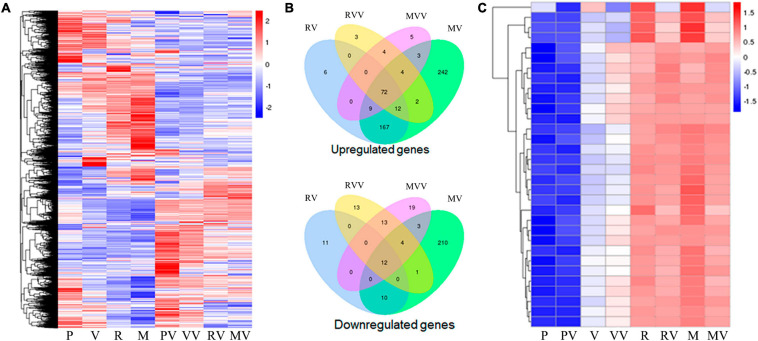
The transcriptome analysis of CARDs transfected PAMs with or without H9N2 infection. **(A)** The heat map of clustered differentially expressed genes (DEGs) in porcine RIG-I and MDA5 transfected PAMs with or without H9N2 infection (0.01 MOI for 12 h). P, PAM mock control; V, pcDNA vector transfection control; R, porcine RIG-I CARDs; M, porcine MDA5 CARDs. The last V denotes H9N2 infection. **(B)** The Venn’s diagrams of several groups of DEGs. RV, RIG-I CARDs vs. pcDNA control; MV, MDA5 CARDs vs. pcDNA control; RVV, RIG-I-CARDs vs. vector control with H9N2 infection; MVV, MDA5-CARDs vs. vector control with H9N2 infection. **(C)** The subcluster with the majority DEGs being ISGs, H9N2 infection causes general upregulations of ISGs, whereas two CARDs induce pronounced upregulations of ISGs; upon H9N2 infection (V), the upregulated ISGs go down.

The CARDs transfected PAMs with or without H9N2 infection were subjected to transcriptome analysis. As showed in the Figure 7, a total of 6431 differential expressed genes (DEGs) were identified and clustered into a heat map (Figure 7A). In the absence of virus infection, the two types of CARDs induced differential gene transcription, while MDA5 CARDs were more effective than RIG-1 CARDs. Specifically, MDA5 CARDs induced 511 upregulated genes and 240 downregulated genes, whereas RIG-I CARDs induced only 266 upregulated genes and 33 downregulated genes (Figure 7B). The results are consistent with the higher signaling activity of MDA5 CARDs. In the presence of virus infection, the two types of CARDs induced comparable gene transcription. Both CARDs induced 97 upregulated genes, whereas RIG-I and MDA5 CARDs induced 43 and 51 downregulated genes, respectively (Figure 7B). We also identified a subcluster, in which 27 out of 32 genes are IFN stimulated genes (ISGs) (Figure 7C). Without CARDs, there are upregulations of ISGs upon virus infection; with two CARDs (which similarly induced ISG upregulation), however, no further upregulation of ISGs was observed in response to H9N2 infection (Figure 7C).

## Discussion

In this study, we investigated the signaling activity and antiviral function of porcine RIG-I and MDA5 N-terminal two CARD domains (CARDs). As the signaling domains, we observed higher signaling activities for the two types of CARDs than the corresponding full-length RIG-I and MDA5. That is as expected based on RIG-I and MDA5 activation mechanisms. Under the steady state, RIG-I CARDs is bound and auto-inhibited by the Hel-2i region of the RIG-I helicase. Upon the binding of RNA ends by RIG-I C-terminal RD, the conformation changes release the auto-inhibition of CARDs (Kell and Gale, 2015). The quiescent MDA5 is also subjected to auto-inhibition, but MDA5 adopts an open and flexible structure (Brisse and Ly, 2019), which renders MDA5 activity higher than RIG-I activity despite its expression being lower than RIG-I.

Similar to full-length MDA5 with higher activity than RIG-I, porcine MDA5 CARDs also exhibits higher activity than RIG-I CARDs. In this case, we do not exactly know the underlying mechanism. However, considering the low amino acid sequence similarities between the RIG-I and MDA5 CARDs, it is very likely that the disparity between two types of CARDs sequences largely determines their structures, signaling activity and protein expressions, which are all different aspects of the intrinsic features of the CARDs proteins. The protein expression level might influence the CARDs signaling activity to certain degree but in a very limited way. Even though the higher expression level of MDA5 CARDs was observed, it is not associated with the higher signaling activity of MDA5 CARDs. One recent study using RIG-I and MDA5 CARDs showed that the oligomerization of the two CARDs utilize distinct mechanisms, with the former in an ubiquitin dependent manner and the latter in a concentration dependent manner (Zerbe et al., 2020). Thus, these internal signaling events should be responsible for the signaling activity of RIG-I CARDs and MDA5 CARDs. Conversely, the analysis of CARDs signaling activity provides another angle to explore the RIG-I and MDA5 activation mechanisms.

Due to the IFN signaling activity, both RIG-I and MDA5 CARDs exhibit broad antiviral functions. The two CARDs not only resist a panel of RNA viruses, including VSV, EMCV, SeV, and influenza viruses, but also resist DNA viruses like HSV-1. It is in contrast with the disparity of full length RIG-I and MDA5 in antiviral functions (Yoneyama et al., 1950; Yoo et al., 2014). Interestingly, regardless the different signaling strengths, the two CARDs showed comparable antiviral activities as well as similar host gene inductions during viral infections. We wonder how the comparable antiviral and regulation of gene transcriptions during viral infection is achieved. It is likely that both RIG-I and MDA5 CARDs have sufficient signaling activities to suppress virus replications. On the other hand, the viruses may have complicated interplay with the CARDs and their signaling pathways, so that the two CARDs both reach a comparable signaling activity.

RIG-I-like receptors are expressed almost in all mammalian cell types, and play key roles in RNA virus sensing and immune responses (Schlee, 2013; Kell and Gale, 2015). Not only anti-RNA virus function, RIG-I, and MDA5 are also implicated in immune responses to other pathogens, including DNA viruses and bacteria (Vabret and Blander, 2013). Pig (*Sus scrofa domesticus*) is an important livestock species but also a valuable biomedical model to study several human diseases, including infectious diseases (Meurens et al., 2012; Mair et al., 2014). However, pigs are subjected to multiple infectious diseases especially those caused by viruses, such as porcine reproductive and respiratory syndrome virus (PRRSV) (Zhao et al., 2019), porcine epidemic diarrhea virus (PEDV) (Cao et al., 2015), classical swine fever virus (CSFV) (Dong et al., 2013), porcine circovirus type 2 (PCV2) (Shi et al., 2020), porcine deltacoronavirus (PDCoV) (Likai et al., 2019), seneca valley virus (SVV) (Li et al., 2018), and african swine fever virus (ASFV) (Ge et al., 2018), etc. Our study on porcine RIG-I and MDA5 CARDs suggests both CARDs possess strong antiviral activities and are thus promising to be developed as common viral vaccine adjuvants or antiviral therapeutics.

## Data Availability Statement

The original contributions presented in the study are included in the article/Supplementary Material, further inquiries can be directed to the corresponding author/s.

## Author Contributions

JZ conceived and designed the experiments. SL, QS, YZ, XJ, JL, YX, and XL performed the experiments. WZ, NC, FM, and JZ analyzed the data. SL and JZ wrote the manuscript. All authors contributed to the article and approved the submitted version.

## Conflict of Interest

The authors declare that the research was conducted in the absence of any commercial or financial relationships that could be construed as a potential conflict of interest.
