# Supplementary material for: Porcine RIG-I and MDA5 Signaling CARD Domains Exert Similar Antiviral Function Against Different Viruses
Source: Front Microbiol. 2021 Jun 11;12:677634. doi: 10.3389/fmicb.2021.677634 (PMC8226225; doi:10.3389/fmicb.2021.677634)
Supplement: Supplementary file 1 [file Table_1.DOCX]

Table 1: Primers for RT-qPCR in this study

| **Primer names** | **Primer sequences** | **Length** |
| --- | --- | --- |
| pIFNβ | F: tgagcattctgcagtacctga | 116 bp |
|  | R: ccggaggtaatctgtaagtctgt |  |
| pISG56 | F: atgggagttggtcattcaaga | 127 bp |
|  | R: caggtgtttcacataggcca |  |
| pTNFα | F: atcgccgtctcctaccaga | 147 bp |
|  | R: tcgatcatccttctccagct |  |
| pβ-actin | F: atgaagatcaagatcatcgcg | 116 bp |
|  | R: tcgtactcctgcttgctgatc |  |
| SIV H9N2 HA | F: aaaccaatgatagggccaa | 204 bp |
|  | R: ttgcactacacagttaccac |  |
| SIV H9N2 M | F: gaccratcctgtcacctctgac | 106 bp |
|  | R: agggcattytggacaaakcgtcta |  |
| HSV1 gB | F: ttctgcagctcgcaccac | 295 bp |
|  | R: ggagcgcatcaagaccacc |  |
| VSV Glycoprotein | F: gaggagtcacctggacaatcact | 121 bp |
|  | R: tgcaaggaaagcattgaacaa |  |
| EMCV Polyprotein | F: tcaccgtgaagtccggcagt | 134 bp |
|  | R: tgtcagacgctgtggcctga |  |
| H1N1 M | F: atcctgtcacctctgactaaggg | 80 bp |
|  | R: tctacgctgcagtcctcgc |  |
| SeV GFP | F: gcaacatcctggggcacaagct | 246 bp |
|  | R: cgcgcttctcgttggggtcttt |  |

Note: p denotes porcine.
